# Effectiveness of Interventions to Improve Digital Health Literacy in Forced Migrant Populations: Mixed Methods Systematic Review

**DOI:** 10.2196/69880

**Published:** 2025-06-25

**Authors:** Achille Roghemrazangba Yameogo, Carole Délétroz, Maxime Sasseville, Samira Amil, Sié Mathieu Aymar Romaric Da, James Plaisimond, Frédéric Bergeron, Sofia Tadlaoui, Patrick Bodenmann, Marie-Pierre Gagnon

**Affiliations:** 1 Faculty of Nursing Laval University Quebec, QC Canada; 2 VITAM - Centre for Research in Sustainable Health Quebec, QC Canada; 3 HESAV School of Health Sciences - Vaud, HES-SO University of Applied Sciences and Arts Western Switzerland Lausanne Switzerland; 4 School of Nutrition Laval University Quebec, QC Canada; 5 Department of Consulting Services Library Laval University Quebec, QC Canada; 6 Department of Vulnerabilities and Social Medicine, Unisanté University Center for General Medicine and Public Health Lausanne Switzerland; 7 Chair of Medicine for Vulnerable Populations Lausanne University Lausanne Switzerland

**Keywords:** digital health literacy, forced migrant populations, refugees, immigrants, interventions, education, systematic review, mixed methods

## Abstract

**Background:**

Digital health literacy (DHL), recognized as a key determinant of health, can influence health and well-being, improve health equity, and reduce health disparities. However, DHL is often limited among forced migrant populations, who usually lack the skills to understand and evaluate health information or to access and use digital health resources appropriately.

**Objective:**

We aimed to (1) identify effective interventions designed to improve DHL among forced migrant populations and (2) categorize and describe the characteristics of interventions that aim to improve the abilities of forced migrants or adapt digital health services to meet the needs and expectations of forced migrant populations limited by low levels of DHL.

**Methods:**

We conducted a mixed methods systematic review according to the PRISMA (Preferred Reporting Items for Systematic Reviews and Meta-Analyses) 2020 guidelines, involving an iterative process among the authors. A medical information specialist assisted in developing a search strategy for the 6 most relevant databases (MEDLINE, Embase, CINAHL, Web of Science, Academic Search Premier, and PsycINFO) and the Google Scholar search engine, covering studies published between 2000 and 2022. Pairs of reviewers selected, individually and independently, titles, abstracts, and then full texts. Data extraction and quality assessment were performed by 2 reviewers and validated by a senior researcher. We used narrative synthesis to provide a comprehensive overview of effective DHL interventions for forced migrant populations, highlighting their success factors.

**Results:**

We identified 1845 studies, of which only 6 (0.33%) were finally selected for narrative synthesis. Studies were excluded due to irrelevance, lack of primary data, or low methodological quality. The analysis revealed a diverse methodological landscape with a predominance of qualitative approaches aimed at understanding the challenges and needs of forced migrants concerning DHL. The main challenges were associated with cultural, linguistic, and practical contexts. Interventions targeted various groups, including older adults, individuals with low literacy or education, and those with limited digital experience. We identified 4 effective educational intervention categories to enhance DHL among forced migrants: education and training; education and social support; enabling and education; and social, educational, technological, and infrastructural support. Overall, most of the studies (5/6, 83%) reported positive results in terms of improving DHL among forced migrants.

**Conclusions:**

This systematic review highlights the importance of improving DHL among forced migrant populations to promote their health and well-being. In addition, it provides comprehensive knowledge about effective interventions conducted with these groups. These findings can inform stakeholders, particularly policy makers, of the need to address low DHL among forced migrant populations. Going forward, these stakeholders need to develop innovative initiatives that rely on holistic approaches and are based on the specific needs of forced migrants to improve equity and health outcomes.

**Trial Registration:**

PROSPERO CRD42022373448; https://www.crd.york.ac.uk/PROSPERO/view/CRD42022373448

**International Registered Report Identifier (IRRID):**

RR2-10.2196/50798

## Introduction

### Background

Forced migration, defined as a nonvoluntary and coerced movement [1] caused by factors such as conflicts, persecution, human rights violations, climate change, natural disasters, and famine [2], is a growing global phenomenon. At the end of 2023, 117.3 million people worldwide had been forcibly displaced as a result of persecution, conflict, violence, human rights violations, and events seriously disturbing public order, including 68.3 million internally displaced persons, 37.6 million refugees, and 6.9 million asylum seekers [3]. These populations face significant challenges in host countries, including limited access to social and health services [4,5] due to precarious migration status, discrimination, language and cultural barriers, and low incomes [6-8]. These obstacles exacerbate their vulnerability and negatively impact their physical and mental health [6,9].

Digital health (or eHealth), based on the use of information and communication technologies for health [10,11], could be a promising avenue to address key challenges in accessing both health information and health care services. This approach proves particularly relevant for forced migrant populations, including internally displaced persons, refugees, and asylum seekers, as well as economic, political, and climate migrants [2,12-18]. These technologies can serve as both tools and sources of health information, enabling users to improve health and reduce health care costs [19,20]. The internet is a cost-effective or free alternative to search for online health information [21]. Accessing health information online can also overcome language barriers by allowing users to use either their native language or the language of the host country [22].

Smartphones and digital platforms are technological solutions that may assist forced migrant populations in navigating complex health systems in host countries [13,18,23]. These digital tools enable these populations, who may be unfamiliar with the organization of health systems in host countries, to locate physicians, clinics, and hospitals [18]. They also facilitate access to health services, including appointment scheduling and geolocation, particularly for services near users’ place of residence [18].

Digital technologies help forced migrants cope with migration-related stressors and challenges in host countries while enhancing their well-being. Their resilience has been consistently linked to social ties [18,24]. Research demonstrates that maintaining connections with community members through digital platforms, along with transnational contact with family via social media and telephone calls, fosters cultural belonging, emotional and social support, and inclusion, all of which help reduce isolation and stress [18,24,25].

Despite the opportunities offered by information and communication technologies, some barriers may hinder forced migrant populations from accessing and using digital health technologies [26,27]. Individual factors such as advanced age, cognitive impairment, and a lack of digital skills exacerbate these challenges [28]. Moreover, the complexity of digital tools, language barriers, and the technical nature of health care systems, particularly the use of specialized medical jargon, can limit access to online health information and services [29-31]. Faced with the flood of information on social media, forced migrants may lack the skills to search for, understand, and assess health-related information, particularly to differentiate between reliable and unreliable health information [32]. Notably, this issue also affects segments of the local population [33]. Finally, in low-resource environments where many forced migrants live, limited or inadequate infrastructure, such as poor internet connectivity and restricted access to digital devices, hinders access to online health information [31,33]. These constraints render even the best digital health interventions ineffective.

Consequently, low digital health literacy (DHL), involving all aforementioned obstacles, is an important issue among forced migrant populations [30,34]. Norman and Skinner [35] define DHL as “the ability to seek, find, understand, and appraise health information from electronic sources and apply the knowledge gained to addressing or solving a health problem.” With the continuous evolution of digital technologies and the increasing complexity of society and health care systems, forced migrants require additional skills, such as the ability to identify information needs, locate credible online health information, and interact effectively with the digital health system, to improve their health and well-being [36]. Several studies [36-39] point to the need to conceptualize DHL by considering dimensions such as context and the interactions between migrants and the digital health system. Low levels of DHL could lead to poor health outcomes [40] and exacerbate disparities in access to digital health services, contributing to health inequalities between communities [33]. Low DHL is not conducive to achieving health equity.

To address these significant challenges, various initiatives aimed at promoting better health behaviors among forced migrant populations have been developed by different stakeholders [14,37,40]. A comprehensive understanding of these interventions to support DHL among forced migrant populations and their effectiveness is essential for policy makers to develop tailored programs and interventions. Reliable information on effective interventions that meet the needs of individuals who have been forcibly displaced can facilitate informed decision-making regarding both the selection of interventions and their implementation. This evidence encompasses various types of interventions (eg, training and education), their characteristics (eg, target population, targeted factors, adaptability to individual specifics, and effectiveness), and the main challenges or conditions necessary for their implementation. However, to our knowledge, limited literature exists on interventions that enhance the DHL of forced migrant populations, which underscores the interest in a mixed methods systematic review.

### Objectives

The overall aim of the mixed methods systematic review was to assess the effectiveness of interventions aimed at improving DHL among forced migrant populations. To achieve this aim, we pursued 2 specific objectives:

- Identify interventions designed to improve DHL among forced migrant populations, including interventions aimed at creating enabling conditions or environments that cater to the needs and expectations of forced migrant populations limited by low levels of DHL, to facilitate their access to, and use of, digital health resources.
- Define the categories and describe the characteristics of these interventions that aim to improve the abilities of forced migrants or adapt digital health services to meet their needs and expectations in the context of limited DHL.

## Methods

### Study Design

We conducted a mixed methods systematic review according to the PRISMA (Preferred Reporting Items for Systematic Reviews and Meta-Analyses) 2020 guidelines for systematic reviews (Multimedia Appendix 1) [41]. We registered the review protocol on PROSPERO (CRD42022373448). The protocol was published in 2023 [42]. The general research question of our review was as follows: how effective are interventions to improve DHL among forced migrant populations, including internally displaced persons, refugees, and asylum seekers, as well as economic, political, and climate migrants?

### Eligibility Criteria

The eligibility criteria of our study were defined based on the PICOS (population, intervention, comparison, outcomes, and study design) model [1,2,43-45] and are described in Multimedia Appendix 2 [1,2,44]. We included studies targeting forced migrant populations, including internally displaced persons, refugees, and asylum seekers, as well as political, economic, and climate migrants. In addition, we included all studies focused on interventions aimed at improving DHL among forced migrant populations. There were no restrictions on the types of studies. We included all quantitative empirical studies, qualitative studies, mixed methods studies, and studies with or without a control group. We considered only studies published in English, French, Italian, Portuguese, or Spanish. We excluded editorials, commentaries, conference abstracts, protocols, and test recordings.

### Search Strategy

A librarian from Université Laval (FB), who specializes in medical information and is experienced in systematic reviews, developed the search strategy in collaboration with the research team. In an iterative process among the authors, we conducted a systematic literature research in the following relevant bibliographic databases: MEDLINE (Ovid), Embase, CINAHL, Web of Science, Academic Search Premier, and PsycINFO. We also used the Google Scholar search engine. This systematic research covered the period from January 1, 2000, to December 15, 2022, because the concept of “digital health” appeared in the early 2000s [46]. The search terms used were based on a combination of 2 key concepts: “digital health literacy” and “forced migrant population.” We developed search terms for each concept from the literature and thesauri. Specific details of the strategies are presented in Multimedia Appendix 3. The search results were collated and uploaded to EndNote 20 (Clarivate) for manual deduplication. The database was then imported into the web-based collaboration tool Covidence (Veritas Health Innovation Ltd) [47], where additional duplicates were removed using the automated deduplication function before study selection began.

### Study Selection and Extraction

Pairs of independent reviewers (ARY, CD, SA, SMARD, JP, and ST) performed the study selection in Covidence [47], where inclusion and exclusion criteria were predefined, first screening abstracts and titles, followed by full-text review of relevant studies. Disagreements were resolved through discussion, and any remaining conflicts were resolved by a third reviewer (M-PG). After study selection, we created a data extraction sheet using Microsoft Excel, piloted it on 3 studies, and refined it for extraction. The final form included study characteristics, health issues, DHL challenges, targeted populations, interventions to improve DHL and their characteristics, and study results. Finally, 2 reviewers (ARY and CD) extracted the data, which were subsequently validated by a senior reviewer (M-PG).

### Quality Data Analysis

Quality assessment was conducted to describe the selected articles and to interpret the data in the synthesis. Two reviewers (ARY and SA) independently assessed study quality using the Mixed Methods Appraisal Tool [48]. Using 5 methodological quality criteria for different designs (qualitative research, randomized controlled trials, nonrandomized studies, quantitative descriptive studies, and mixed methods studies), this tool allows the simultaneous assessment of different types of studies (eg, qualitative, quantitative, or mixed methods). To achieve a congruent understanding of the criteria, a pilot test was conducted before the independent quality assessment by the evaluators. Differences in judgment were discussed, where necessary, to reach a consensus.

### Data Synthesis and Analysis

We used narrative synthesis [49] as a method, regardless of the type of study (eg, qualitative, quantitative, or mixed methods), to provide a descriptive synthesis of the results of the included studies. We report the study characteristics and methods in the Results section using a quantitative approach. We report the qualitative analysis results in the form of themes. The relevant data from the included studies have been summarized and analyzed in narrative format, with findings grouped or themed wherever applicable as follows: (1) descriptions of target groups, level of intervention implementation, and DHL challenges among forced migrant populations; (2) interventions to improve DHL; and (3) the effectiveness of interventions to improve DHL.

## Results

### Selection Process

**Figure 1:**
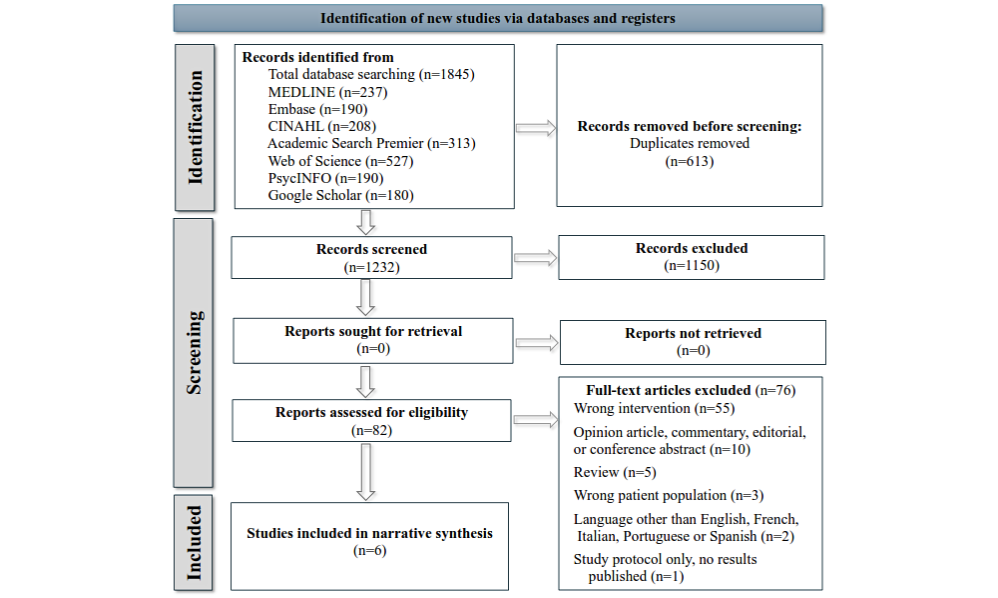
PRISMA (Preferred Reporting Items for Systematic Reviews and Meta-Analyses) 2020 flow diagram showing the number of studies identified, screened, assessed for eligibility, and included in the narrative synthesis.

The bibliographic searches identified 1845 publications. After removing duplicates, 1232 publications were screened, of which 613 (49.76%) were excluded based on titles or abstracts because they did not meet the inclusion criteria. At the end of this first selection, 82 studies were selected for a review of the full text. At the end of the full-text review, we excluded 76 (93%) of the 82 studies, thus leaving 6 (7%) studies [50-55] included in our systematic review. The PRISMA flow diagram showing the selection process is presented in Figure 1.

### Characteristics of Included Studies

The main study characteristics and methods are presented in Table 1. The 6 studies were published in various journals. The oldest was published in 2017 [51] and the most recent in 2021 [55]. Of the remaining 4 studies, 1 (25%) was published in 2018 [54], followed by 2 (50%) [50,52] in 2019, and finally 1 (25%) in 2020 [53]. Of the 6 studies, 2 (33%) were conducted in the United States; 2 (33%) in Europe (n=1, 50% in Greece and n=1, 50% in Spain); 1 (17%) in Australia; and 1 (17%) in the Middle East, specifically in Israel.

Regarding the methods used, half of the included studies (3/6, 50%) applied qualitative approaches [51,52,54]. Of these 3 studies, 1 (33%) used face-to-face interviews conducted with Ethiopian immigrants in Israel to explore their experiences of using a website [51]. Ripple effects mapping, a participatory qualitative assessment method, was used to assess the effects of a peer language navigator (PLN) program in Anchorage, Alaska [52]. The study by Millard et al [54], conducted in Perth, Western Australia, combined qualitative methods (participant observation, social network mapping, and ethnographic and life history interviews) to understand the important role of digital citizenship, including the ability of older migrants to participate in society using digital technologies and platforms to improve their well-being.

**Table 1:** Characteristics of included studies.

Study	Objective	Country	Study population (sample size)	Study design	Challenges of DHL^a^ for target population
Fernández-Gutiérrez et al [50], 2019	To evaluate the effectiveness of an mHealth^b^ intervention to improve the cognitive and social skills that enable migrants to access and use health services	Spain	Immigrants aged 18-65 y of non-Spanish nationality from sub-Saharan Africa, China, eastern Europe, Morocco, and South America (n=93)	Quasi-experimental design: 1 group only, pretest-posttest measurements	Measure of health literacy: the mean HLS-EU-Q16^c^ score was 9.55 (SD 4.35), corresponding to a problematic level of health literacy (with scores ranging from 0 to 16 points, this questionnaire establishes 3 levels of health literacy: inadequate, 0-8; problematic, 9-12; and sufficient, 13-16)
Guttman et al [51], 2017	(1) To introduce Ethiopian immigrants, mainly those with low or no literacy, both in Hebrew and Amharic, to using a new health information website with an interface that does not require reading skills; and (2) to explore how they would respond to the experience of using the website, its relevance to their lives, and current and potential barriers they might anticipate for future use	Israel	Israeli Ethiopian immigrants, cultural and linguistic minorities facing sociocultural and health disparities (n=225)	A qualitative study with two phases: (1) health topic selection (discussions with staff of Tene Briut, a nongovernmental organization that delivers health care services throughout Israel, and interviews with Ethiopian community members and practitioners), and (2) a qualitative descriptive study (face-to-face interviews with Ethiopian immigrants to learn about their observations and an analysis and discussion of the findings)	No measure used; low or no literacy skills to browse online health information
Johnson et al [52], 2019	To develop and implement a peer language navigator program to improve health literacy in immigrant communities	United States	Immigrant and refugee communities in Anchorage, Alaska (NR^d^)	Ripple effects mapping: a participatory method of qualitative evaluation of the program	No measure used; low health literacy level because of limited English proficiency; most of the residents were unfamiliar with where or how to obtain health care services
Kim et al [53], 2020	To develop a website that helps populations with LEP^e^ increase health literacy and improves health care service access	United States	Refugees from the Karen ethnic community with LEP and low health literacy (n=22)	A mixed methods study comprising three phases: (1) a needs assessment with community leaders and service providers (qualitative in-depth interviews); (2) the development of content from credible sources, with each item tested using multiple readability assessments (quantitative study); and (3) a revision of each item to lower the reading level, followed by retesting for readability (quantitative study)	No measure used; LEP issues; information in English had limited readability; low or no skills in researching health information and navigating the health care system in English
Millard et al [54], 2018	To understand the increasingly important role of digital citizenship (the ability to participate in society using digital technologies and platforms) in supporting the well-being of aging migrants	Australia	Older adult migrants in Perth, Western Australia, taking part in an “Internet cafe” initiative to improve their internet skills and well-being (n=15)	Qualitative study (participant observation, social network mapping, and ethnographic and life history interviews)	No measure used; age-based digital divide: most of the participants had little or no experience using digital communication technologies before coming to the internet cafe
Riza et al [55], 2021	To test the potential of using an electronic algorithm in low-resource primary care settings with the help of health professionals in temporary settlements to help improve the health status of migrants and refugees, increase their health literacy, and facilitate their integration into the host communities	Greece	Migrants and refugees residing in reception and identification centers (n=82)	Quantitative descriptive study	No measure used; cultural, linguistic, and practical obstacles; most of the participants had difficulty understanding medical information and did not know where to seek medical help for a specific health problem

^a^DHL: digital health literacy.

^b^mHealth: mobile health.

^c^HLS-EU-Q16: European Health Literacy Survey Questionnaire.

^d^NR: not reported.

^e^LEP: limited English proficiency.

Of the 6 included studies, 2 (33%) used quantitative methods. The first tested the use of an electronic algorithm to help migrants and refugees improve their ability to understand medical information, assess health status, and navigate the health system [55], while the second examined the DHL level of migrants and assessed the effectiveness of a mobile health intervention aimed at improving the cognitive and social skills that enable access to, and use of, health services [50]. Finally, only 1 (17%) of the 6 included studies used mixed methods, combining interviews to assess the needs of the community and quantitative methods to test and validate the readability of website content [51].

### Descriptions of Target Groups, Level of Intervention Implementation, and DHL Challenges Among Forced Migrant Populations

Table 2 shows that the studies were conducted at the individual, community, or societal level or simultaneously at all 3 levels. The target populations were mainly immigrants and refugees. The sample sizes varied from 15 [54] to 225 [51] (Table 1).

The populations under review faced some challenges related to DHL (Table 1). Only 1 (17%) of the 6 studies measured the DHL level among participants before the implementation of the intervention [50]. The participants in the remaining studies (5/6, 83%) exhibited personal characteristics that defined their low level of DHL.

At the individual level, the study by Riza et al [55], which was the only one in this category, individually and directly targeted migrants and refugees residing in reception and identification centers in Greece who faced cultural, linguistic, and practical barriers. Most of them had difficulty understanding medical information and did not know where to seek medical help for a specific health problem.

At the community level, 3 studies were identified. The study by Fernández-Gutiérrez et al [50] specifically targeted population groups of immigrants of non-Spanish nationality from sub-Saharan Africa, China, eastern Europe, Morocco, and South America residing in a particular region (the border area of southern Spain). This was the only study that included a preintervention assessment of participants’ DHL. Health literacy was measured using the European Health Literacy Survey Questionnaire (HLS-EU-Q16), which assigns scores ranging from 0 to 16 points. This questionnaire classifies health literacy into 3 levels: inadequate (0-8), problematic (9-12), and sufficient (13-16). The results indicated a problematic level of health literacy among these groups, with an average HLS-EU-Q16 score of 9.55 (SD 4.35). The study by Johnson et al [52] specifically targeted immigrant and refugee populations in Anchorage, Alaska. Most of these people were newcomers and had little or no English language skills, which is a handicap in understanding online health information, navigating the health system, and accessing appropriate care. Kim et al [53] targeted immigrants and refugees with limited English proficiency (LEP), specifically the Karen ethnic community from Myanmar (formerly Burma). This group faced problems with readability, defined as the ability to find the right health information in an accurate, reliable, and easy-to-understand format, which indicates the specific DHL needs of a particular community.

**Table 2:** Interventions that aimed to improve digital health literacy (DHL).

Intervention categories and studies			Action level		Target population		Interventions to improve DHL
**Education and training**							
	Fernández-Gutiérrez et al [50], 2019	Community		Immigrants in Spain from sub-Saharan Africa, China, eastern Europe, Morocco, and South America		e_SaludAble, a culturally appropriate mobile app containing health information, was developed for migrants in Spain. It featured a main menu in 6 languages, with 6 sections arranged in a scrolling tree structure designed to promote access and facilitate navigation of the sociomedical system to promote and maintain well-being. Participants were trained in their mother tongue on the app’s basic content and how to use it. The aim was to improve the cognitive and social skills that enable migrants to access and use health services.	
	Guttman et al [51], 2017	Individual, community, and societal		Israeli Ethiopian immigrants, cultural and linguistic minorities, facing sociocultural and health disparities		A website was created to present health information through videos in Amharic. It opened with a video explaining the purpose of the website and how to use it. The website featured an audiovisual interface with images and voice guidance to enable simple navigation among health topics without the need for reading or typing skills. Topics could be heard aloud when the cursor hovered over a picture or title. The hierarchical design enabled users to navigate from a general topic to specific topics (eg, symptoms explained using cultural metaphors). The topics included women’s health, nutrition, and diabetes.	
**Education and social support**							
	Johnson et al [52], 2019	Community		Immigrant and refugee communities in Anchorage, Alaska, United States		The peer language navigator program aimed at enhancing health literacy among immigrant and refugee communities in Anchorage, Alaska. Individuals from these communities were trained about health and wellness topics as well as how to obtain health information from reliable online sources. Peer language navigators then shared health information resources with their respective communities.	
	Riza et al [55], 2021	Individual		Migrants and refugees residing in reception and identification centers in Greece		An IT-based algorithm was designed to assess the health status of migrants and refugees residing in reception and identification centers in Greece. This intervention involved the use of portable electronic devices, such as tablets, to administer a structured questionnaire that collected data on various health-related aspects, including health literacy, mental health, vaccination history, lifestyle habits, and the presence of diseases. Upon completion of the questionnaire with the help of health professionals, a report could be downloaded highlighting the health issues needing further attention, and guidance was provided on where to find help (health care system navigation information).	
**Enabling and education**							
	Kim et al [53], 2020	Community		Refugees from the Karen ethnic community with limited English proficiency and low health literacy in 1 geographic location in the United States		A health information website was specifically developed to assist populations with limited English proficiency in increasing their health literacy and improving access to health care services. The website was characterized by simplified content, that is, information was presented in a clear, easy-to-read, and easy-to-understand manner. It served as an educational and informative resource aimed at providing accessible health information tailored to the needs of immigrants and refugees, specifically the Karen refugee community from Myanmar (formerly Burma)	
**Social, educational, technological, and infrastructural support**							
	Millard et al [54], 2018	Individual, community, and societal		Older adult migrants in Perth, Western Australia		An internet cafe that provided digital literacy training specifically for older adult migrants was established in Perth, Western Australia. It was based on a social learning approach where participants could develop their digital skills at their own pace by using the internet and digital technologies, guided by the staff of the organization and fellow patrons (peer learning). By fostering a community of practice, the intervention aimed to improve participants’ confidence, autonomy, and access to social networks and essential services through technology.	

Finally, in 2 (33%) of the 6 studies, interventions were conducted simultaneously at the individual, community, and societal levels. The intervention in the study by Guttman et al [51] directly targeted Ethiopian immigrants in Israel with little or no reading skills, suggesting difficulties in accessing online health information. The researchers also involved Ethiopian immigrant organizations and promoted the use of the website in community contexts. At the societal level, the website was designed to link national information about the specific needs of the immigrant community. In the study by Millard et al [54], the intervention, an “Internet café,” directly targeted older migrants to improve their essential digital skills. Most of these migrants had little or no experience in the use of digital communication technologies before coming to the internet cafe. The intervention functioned as a community of practice where participants learned from each other in a supportive social environment, helping to bridge the digital divide within society.

### Interventions to Improve DHL

To improve DHL among targeted forced migrant populations, we identified 4 categories of educational interventions: training; social support; empowerment (enablement); and educational, technological, infrastructural, and social support (Table 2).

In the category of training, 2 studies were identified. The first intervention involved the use by groups of immigrants in Spain of a culturally appropriate mobile app (e_SaludAble) containing health information [50]. e_SaludAble featured a main menu in 6 languages, with 6 sections spread across a drop-down tree structure designed to facilitate access and navigation through the social and health system to promote and maintain wellness. Thus, depending on the mother tongue and nationality, groups of participants were trained and sensitized to the app’s basic content and its use. The goal was to improve immigrants’ cognitive and social skills related to accessing and using health services. The second intervention involved the creation of a website that presented health information through videos in Amharic [51]. It opened with a video explaining the purpose of the website and how to use it. The website featured an audiovisual interface with images and voice guidance, allowing for simple navigation among health topics without the need for typing or reading skills. Designed to be accessible, the website aimed to promote access by individuals to online health information and was considered a community resource.

Social support was the second category, with 2 identified interventions. The PLN program [52] aimed to improve skills to understand and evaluate health information for immigrant and refugee communities in Anchorage, Alaska. The program trained members of these communities on health and well-being issues, as well as how to obtain health information from trusted online sources. These trained individuals (PLNs) then served as “navigators,” sharing relevant health information with their respective communities and helping their peers access health care and services. The second intervention was an electronic algorithm designed in the form of a questionnaire to assess health status [55]. It aimed to help migrants and refugees residing in reception and identification centers in Greece conduct a health self-assessment by answering a personalized questionnaire with the help of health professionals. This tool offered individuals specific information about their health status, health problems, and tips on where to seek help in the health care system.

The third category was empowerment (enablement), aiming to strengthen the capacities and autonomy of individuals and communities by making resources (both individual and collective) available and accessible [56]. The goal was to enable these individuals to take control of their lives, decisions, and environments. As such, the intervention in the study by Kim et al [53] included the development of a health information website aimed at providing accessible and adapted information to immigrants and refugees. The content was simplified to present clear, easy-to-read, and easy-to-understand information. This website was specifically designed to help immigrants and refugees with LEP, particularly the Karen ethnic community, and aimed to increase their competence to understand health information, navigate the health system, and make informed decisions about their health.

The final intervention category was educational, technological, and infrastructural support, for which we identified 1 intervention [54]. This intervention involved the creation of an internet cafe that helped older immigrants in Perth, Western Australia, improve their essential digital skills. It was based on a social learning approach, which allowed participants to progress at their own pace and learn from each other. Older immigrants were guided by the organization’s staff and peers in the community in their learning. By fostering a community of practice, the intervention aimed to improve confidence, maintain support networks, facilitate access to essential services, and support older immigrants’ autonomy to participate in society using digital technologies and platforms and improve their well-being.

### Effectiveness of Interventions to Improve DHL

We evaluated all interventions presented in the 6 studies aimed at improving DHL among forced migrant populations. As shown in Table 3, overall positive results were reported in 5 (83%) of the 6 studies [50,52-55], while 1 (17%) study had mixed results [51].

In the training category, 2 culturally appropriate interventions were identified, with differing outcomes. The intervention in the study by Fernández-Gutiérrez et al [50] was successful. The results showed that health literacy improved significantly after the intervention, with the average HLS-EU-Q16 score increasing from 9.55 (SD 4.35), considered problematic, to 14.03 (SD 2.68), deemed sufficient. The training and awareness-raising activities significantly improved participants’ required cognitive and social skills for using the culturally appropriate mobile app (e_SaludAble), enabling them to access health information and health services. These results were valid for both men and women as well as participants of all nationalities, except for the Chinese group. The authors attributed this exception to cultural factors, noting that decision-making among Chinese individuals is often influenced by family, peer groups, or community leaders [50]. As a result, the performances of Chinese participants yielded results that were different from those of other nationalities.

The results of the study by Guttman et al [51] were mixed. Most of the participants reacted enthusiastically to their experience of using the website. Some were even overwhelmed by the new opportunity to access detailed health information. Others emphasized that the site addressed their concerns, the language was respectful, and the images represented their culture. By contrast, some participants had a negative experience. Some felt that the site was too difficult to use and that they needed support and training. Other participants felt that the website was not intended for them because they were too old to learn and did not think they could use it.

**Table 3:** Main study results.

Study	Main outcome	Secondary outcomes
Fernández-Gutiérrez et al [50], 2019	Positive results: a significant improvement in individuals’ health literacy was observed after the intervention, with the mean HLS-EU-Q16a score increasing from a “problematic” 9.55 (SD 4.35) to a “sufficient” 14.03 (SD 2.68; z=−6.256, P<.001).	The differences between pretest and posttest intervention scores were statistically significant for both men and women and for participants of all nationalities, except for the Chinese group.
Guttman et al [51], 2017	Positive results: the experience was generally rewarding, and most of the participants reacted with enthusiasm and excitement. Some were even overwhelmed by the new opportunity to access detailed health information. Other participants expressed that the website was “designed for them” and addressed their concerns, the language used was respectful, and the images represented their culture. Negative results: the experience was also marked by apprehension, even frustration, about future use. Participants with low or no literacy skills felt that the website was too difficult to use and expressed the need for support and training. Others felt that the website was not intended for them because they were too old to learn and did not think they could use it.	The analysis of participants’ apprehensions about using the website generated several conceptual developments: (1) the addition of culturally focused elements to the Technology Acceptance Model; (2) the identification of users’ needs according to their conceptions of their capabilities; (3) the highlighting of ways in which an information website can offer sociocultural benefits in addition to providing relevant information, including intergenerational communication; and (4) the proposal for the integration of the website into the wider communication infrastructure of a linguistic minority.
Johnson et al [52], 2019	Positive results: the PLNb program has proven its effectiveness in providing understandable health information to hundreds of new English language learners in Anchorage, Alaska, while guiding them to reliable health and wellness resources that they can use for themselves, their families, and their community.	PLNs improved their health literacy, enabling them to become a credible and useful resource for others. At the end of the training, >90% of the PLNs reported feeling confident or very confident in their ability to find health information through an internet search. PLNs became leaders in their communities. The PLNs’ contact logs showed that they communicated with many people in their respective communities. The most recent cohort, made up of 5 PLNs, provided health information to >150 people over 6 mo. The PLNs’ work reached not only individuals but also community organizations and events.
Riza et al [55], 2021	Positive results: the application of an electronic algorithm helped to identify gaps in the understanding of health concepts, such as understanding medical information in leaflets, and it provided study participants with useful links to tools to increase their knowledge in several thematic areas. Using the web map, the participants had the opportunity to locate points of care that they could access to seek professional help for a medical issue.	67.1% of the study participants encountered difficulties in understanding medical information, and 57.3% did not know where to seek medical help for a specific health problem. Four main areas of health problems, where the assistance of a health care professional was required, were identified: (1) mental health concerns, (2) vaccinations, (3) obesity, and (4) dental hygiene. The “Roadmap and Toolbox” section of the project website gave respondents access to numerous resources and tools to enhance their knowledge in several thematic areas.
Kim et al [53], 2020	Positive results: the average reading level of the original 99 topics was assessed at 10.84 (SD 3.26). After revisions, the authors were able to lower the reading level to 8.56 (SD 2.96), corresponding to a drop of approximately 2 grade levels on average.	Online health information platform: 99 health topics (40 related to hospitals, 37 to primary health services, and 22 to Medicaid) were identified, and appropriate content was developed. Most of the feedback received during the postdevelopment evaluation was positive, particularly concerning the health information available on the site. However, average scores could not be reduced below the recommended level for grade 7, as recommended by leading organizations, such as the American Medical Association and the National Institutes of Health.
Millard et al [54], 2018	Positive results: with appropriate educational, technological, infrastructural, and social support, digital literacy for older adult migrants can significantly enhance their ability to maintain and expand dispersed support networks, as well as to engage socially and access health services. The “Internet cafe,” which fostered social learning environments and communities of practice, contributed to the development of digital literacy among older adult migrants, enabling them to benefit from greater autonomy, that is, increased motivation and the ability to use the internet for information and communication.	Older migrants want to connect with members of their support networks, whether local or remote, online or offline. When they have access to the tools and skills of new technologies and the internet, older adult migrants benefit from increased direct access to their social networks, as well as to information, services, and personal interests, even at a distance. As they become more digitally literate, older adult migrants feel a greater sense of autonomy and social participation. Social learning environments, which function as communities of practice, are proving to be effective means of developing digital literacy and digital citizenship. These communities of practice can be supported by qualified ICTc and social service professionals, in collaboration with individuals and other members of their respective care networks.

^a^HLS-EU-Q16: European Health Literacy Survey Questionnaire.

^b^PLN: peer language navigator.

^c^ICT: information and communication technology.

All interventions in the other categories were successful in improving DHL among forced migrant populations. The PLN program in the study by Johnson et al [52] demonstrated a beneficial effect in providing understandable health information to hundreds of migrants who were new English learners. This program guided them to reliable health and well-being information, enabling them to use it to make informed decisions to manage their health as well as that of their families and communities. The PLNs improved their own skills in understanding health information and navigating the health system, allowing them to serve as credible and useful resources for others. More than 90% of the PLNs said that they were confident or very confident in their ability to find health information through an internet search.

The study by Riza et al [55] demonstrated the effectiveness of an electronic algorithm as a tool to improve health literacy. It helped users to understand health information and identify areas that needed attention, such as mental health support, advice on vaccination, weight management (obesity), and dental care. In addition, the results highlighted gaps in the health status of immigrants and refugees. Finally, the algorithm made it possible to provide health information and resources tailored to their personal needs (useful links), strengthening their ability to make informed decisions to better manage their health.

Positive results were also observed in the study by Kim et al [53] about making a website more accessible for migrants with LEP. The average reading level of the information was lowered from 10.84 (SD 3.26) to 8.56 (SD 2.96), that is, approximately 2 grade levels lower on average. The information on the website was accessible, easy to read, and easy to understand. Most of the feedback received during the postdevelopment evaluation about the understanding of information by immigrants and refugees with LEP was positive.

The intervention in the study by Millard et al [54] was also successful. The study demonstrated that the internet cafe, which fostered social learning environments and communities of practice, contributed to the development of digital literacy and digital citizenship among older migrants, allowing them to maintain their support networks, access essential information and services, and enjoy greater autonomy and social participation. The internet cafe improved older migrants’ motivation and ability to use the web for information and communication purposes, such as connecting with members of their support networks, whether local or remote, online or offline.

### Quality Assessment of the Included Studies

Table 4 presents the quality assessment of the included studies. Although specific to each approach, whether qualitative or quantitative, 5 criteria were used to assess study quality. Overall, most of the studies met either 80% (4/6, 67%) [51-54] or 100% (2/6, 33%) [50,55] of the quality criteria. However, some limitations were noted in these studies that do not call into question the quality of the results.

**Table 4:** Quality of included studies.

Study	Quality criteria, n (%)
Fernández-Gutiérrez et al [50], 2019	5 (100)
Guttman et al [51], 2017	4 (80)
Johnson et al [52], 2019	4 (80)
Kim et al [53], 2020	4 (80)
Millard et al [54], 2018	4 (80)
Riza et al [55], 2021	5 (100)

In the qualitative studies, the main limitations pertained to the generalizability of the results. These studies targeted communities in specific contexts, such as Ethiopian migrants in Israel [51], migrants and refugees in Alaska [52], and older adult migrants in Western Australia [54]. Additional limitations were related to sample sizes and participant selection methods, which restricted the representativeness of the results [52,54]. Some researchers [51] did not have access to the participants’ responses in their original language, and the participants did not have enough time during the interview to review all topics and make their selections.

Regarding the quantitative studies (2/6, 33%) [50,55], selection biases were noted. The study by Riza et al [55] also reported difficulties in accessing certain categories of targeted populations that were difficult to reach. In addition, the study by Fernández-Gutiérrez et al [50] highlighted limitations associated with the use of the HLS-EU-Q16: the reduction in the number of questionnaire items from 47 to 16 led to a considerable loss of information.

Finally, the mixed methods study by Kim et al [53] focused on a specific group in a single geographic location, that is, individuals with LEP from the Karen refugee community from Myanmar (formerly Burma) residing in 1 region of the United States. Therefore, the results from this study cannot be generalized.

## Discussion

### Principal Findings

This systematic review explored the effectiveness of interventions aimed at improving DHL in forced migrant populations. The importance of gaining DHL skills is well established for populations with low DHL, such as forced migrants [57-59]. These skills enable forced migrant populations to promote their health and well-being. These people can use digital tools, such as the internet, smart and connected devices, mobile apps, and online platforms, to access relevant health information, care, and health services via digital or remote means. In the age of digital technology and artificial intelligence, DHL is a major determinant of health across populations [60]. Due to the context of the vulnerability of forced migrants [42], it is essential to support the development of their skills to search; find; understand; and, especially, critically evaluate health information. This also includes the ability to apply or create health information for communicating and interacting with the health system to maintain or improve quality of life. Thus, these populations represent important targets for the development of interventions that support their DHL, as well as for evaluating the effectiveness of these interventions.

Existing literature on the subject is relatively recent and rare but has been growing in recent years [61]. This period coincided with the advent of the COVID-19 pandemic, which accelerated the global shift toward the use of digital tools to access information, care, and health services [30,62]. The pandemic acted as a catalyst for the rapid adoption of digital health technologies [61,63], which has likely contributed to the increase in studies and initiatives aimed at strengthening digital health skills across populations, particularly forced migrants.

The analysis of the 6 included studies showed methodological diversity and richness. The DHL of forced migrant populations is a sensitive topic that requires special attention to individuals’ cultural and social contexts, personal characteristics, and environments [40]. The qualitative approaches used focused on participants’ experiences, which allowed for an in-depth understanding of their contextual and cultural challenges and specific DHL needs [64,65]. In general, qualitative approaches capture the complex realities of populations experiencing vulnerability, which are often overlooked in quantitative studies [66]. Understanding the specific challenges and needs made it possible to tailor interventions to the specific realities of forced migrant populations. At the same time, quantitative studies, such as the one by Fernández-Gutiérrez et al [50], provided empirical data on the health literacy levels of migrants and refugees, data that are essential for assessing intervention effectiveness [67].

The geographic diversity of the studies, spanning the United States, Australia, and European and Middle Eastern countries, also highlighted varied cultural contexts that influence the results and methods of interventions. These countries have been among those that have received the highest numbers of forced migrant populations worldwide in recent years [68]. Adapting interventions to the cultural context of individuals is essential for their success in these host countries [69]. Although the results of the various studies are promising in terms of transferability, it is crucial to consider cultural differences and specific local conditions when adapting interventions. The results of our systematic review show that the main target groups identified varied greatly. These groups included older adults, people with lower levels of literacy or education, and those with little or no experience with digital tools. The challenges related to DHL were therefore varied and mainly related to cultural, linguistic, and practical contexts. The individual characteristics of these groups made them more vulnerable and made it more difficult for them to access health information and services. Previous research indicates that age, level of education, experience, and digital literacy influence the DHL level of individuals [33,64,70]. As a result, these populations faced significant barriers to DHL, highlighting clear DHL needs among these immigrant and refugee groups.

Of the 6 included studies, only 1 (17%) [50] conducted a preintervention assessment of DHL needs, highlighting a lack of systematic assessments of digital health skills in this context [71]. The results showed that forced migrant populations often have problematic levels of DHL, highlighting the importance of designing interventions tailored to the specific needs of these groups. The categories of interventions identified (ie, education and training; education and social support; empowerment and education; and educational, technological, infrastructural, and social support) to improve DHL among forced migrant populations reflect integrated and multidimensional approaches. Each type of intervention has its strengths and weaknesses, illustrating the complexity of the needs of forced migrant populations. Overall, the various interventions were successful. However, those that target certain categories, such as older migrants and migrants with less education, require increased attention and more robust approaches. The effectiveness of the interventions in most of the studies (5/6, 83%) was found to be contingent upon their cultural and linguistic adaptation to individuals’ personal characteristics and specific DHL needs. For some researchers [65,69,72], adapting interventions to the cultural context of populations is essential for their success, as it is a form of communicating respectfully; honoring cultural values; and tailoring actions considering the needs of individuals as well as the diversity of contexts, especially in contemporary societies. This approach not only recognizes the unique challenges faced by forced migrant populations but also leverages their cultural strengths to foster resilience and improve their DHL. Our research has identified a multitude of cultural and linguistic adaptation considerations.

The first consideration mentioned in the studies was the use of appropriate language and medical terms in the design and implementation of interventions. This is an essential aspect of cultural adaptation [73]. Therefore, linguistic and cultural differences in understanding information and accessing health services are major barriers to individuals’ engagement, involving cognitive, affective, behavioral, and contextual dimensions [69]. Our research has demonstrated the effectiveness of education and training. This category included organizing training and information sessions conducted in English [52,53], the mother tongue [50], or local or preferred language [51] of forced migrant populations. In addition, in some interventions such as website creation [52], the reading level of medical information was lowered to support populations with LEP in their empowerment and education. As a result of the different interventions, the cognitive (understanding and evaluating information as well as informed decision-making) and social (communication, collaboration, and social support) skills of the targeted forced migrant populations improved significantly. These results are consistent with those of Michie et al [43,44], who found that education, training, and empowerment interventions improve the abilities, opportunities, and motivation of individuals to change their behaviors, particularly in the context of managing their health and well-being, including through the use of digital tools. Language is not limited to a simple vector of communication; it also conveys cultural values, beliefs, and norms that influence how individuals perceive and interpret messages, and how they behave [69]; thus, more attention should be paid to medical terminology choice. In sum, an intervention that considers the specific language and language needs of individuals can play a crucial role in improving their DHL level.

The second consideration discussed in the included studies was the social support provided by family, peers (competent community members), professionals, and community organizations to forced migrant populations. Social support is often cited as an effective method to strengthen individual and community engagement as well as networks [74]. In the reviewed studies, social support helped build social capital, which was essential for individuals’ well-being and engagement. Forced migrant populations participated in a collaborative learning environment with peers, professionals, and community organizations, which provided an opportunity to share knowledge and experiences to address DHL challenges [75]. In addition, institutional and organizational efforts were made to address gaps in access to relevant health information. Community peers or family members acted as mediators between forced migrant populations and existing resources, allowing information and tools to be adapted to the specific needs of these groups, considering cultural, linguistic, and technological barriers [76].

The third consideration relates to the level of implementation of the interventions presented in the included studies, a factor that directly influenced the first 2 considerations. The reviewed studies identified 3 levels of intervention implementation: individual, community, and societal. Of the 6 studies, 2 (33%) [51,54] combined these 3 levels, highlighting the specificities of interventions to improve DHL among forced migrant populations. The importance of a multilevel approach to supporting DHL among forced migrant populations is clear; therefore, it is essential that interventions are tailored and contextualized, considering their realities and needs [76,77]. This strategy has the immense advantage of offering holistic and integrated interventions, considering the key dimensions that influence the DHL of forced migrant populations in host countries.

Thus, this approach has broader implications, that is, implications that go beyond the individual framework, encompassing the community, institutions, and society [78]. Such interventions allow forced migrant populations to better understand their options, access community and institutional services, and improve their engagement and DHL.

### Strengths and Limitations

This study has several strengths, including the systematic analysis of the diverse approaches and methods adopted in the 6 included studies, which provided insights into interventions to improve the DHL of forced migrant populations. The variety of approaches and methods used allowed for a rich and nuanced understanding of the challenges faced by these groups as well as the effectiveness and impact of the interventions. The interventions were found to be culturally and linguistically appropriate to forced migrant populations, thereby enhancing their effectiveness. In addition, several studies demonstrated positive results in improving DHL, highlighting the concrete impact of these initiatives on improving the health and well-being of participants.

However, the methods adopted had significant limitations. The heterogeneity in study settings and designs limited the possibility of combining results. The focus on forced migrant populations may have excluded relevant interventions targeting other groups that could be relevant to improving DHL in forced migrants.

Given that the reviewed studies focused on specific populations in particular contexts, generalizing the results to other groups or regions could prove problematic. Data were lacking from certain regions, such as Africa and South America. Moreover, most of the studies (5/6, 83%) did not include comparison groups or had short-term, if any, follow-ups, making it difficult to assess the long-term efficacy and comparative effectiveness of the interventions. In addition, some of the studies suffered from selection bias (2/6, 33%) [52,55] and limited sample sizes (1/6, 16%) [54], which may affect the robustness of the conclusions. Finally, most of the studies (5/6, 83%) did not systematically measure the DHL level among participants before intervention implementation, which may limit the ability to accurately assess the effectiveness and impact of the interventions.

### Future Directions

For future research, it is important to develop effective, tailored, and acceptable interventions for forced migrant populations. These interventions should be centered on their needs and grounded in evidence, while also considering their values, preferences, and digital health capabilities.

Studies should adopt approaches that enable a deep understanding of the specific challenges faced by forced migrant populations, particularly regarding DHL. To achieve this, it is recommended to include diverse and representative samples from different countries, cultures, languages, and educational levels. This approach will help clarify variations in DHL and support the design of tailored interventions. Ethically, studies must adopt inclusive approaches, considering the specific barriers faced by groups of forced migrant populations experiencing vulnerability, such as older adults, individuals with lower educational levels, those with limited financial resources, people with minimal digital experience, or those living in low-resource settings (eg, rural areas). Furthermore, it is crucial to consider the social, economic, cultural, and environmental determinants of health that influence DHL. An intersectional analysis of these factors would provide a better understanding of inequalities and help propose more equitable solutions.

The use of mixed methods is recommended to generate reliable knowledge and support informed decision-making in the development of tailored interventions. Qualitative approaches can offer an in-depth analysis of individual experiences and the challenges faced by forced migrant populations in host countries, while quantitative methods will allow for the study of the impact and effectiveness of interventions, particularly by measuring DHLs level before and after interventions. In addition, longitudinal follow-up with participants could strengthen the assessment of the long-term impact of these interventions.

To maximize their impact on a larger scale, interventions should adopt holistic and multilevel approaches, integrating actions at the individual, community, and societal levels:

- At the individual level, this involves, for example, strengthening digital health skills through education and tailored, accessible training. By integrating feedback mechanisms and continuous learning, interventions can ensure that forced migrant populations remain competent and confident in their use of digital health technologies.
- At the community level, partnerships with local organizations and community leaders can facilitate support for forced migrant populations while fostering their ownership of the interventions. Community-based digital literacy programs can create peer support networks, enhancing long-term adaptability and confidence in using digital tools.
- Finally, at the societal level, inclusive public policies and accessible digital infrastructure are necessary to create an enabling environment for improving DHL. Policies should prioritize the development of user-friendly, culturally adapted digital solutions, while ensuring continuous access to updated resources, enabling forced migrant populations to remain proficient in the use of digital health tools over the long term.

By adopting these recommendations and strategies, future research will be better equipped to address the DHL needs of forced migrant populations while overcoming the methodological and contextual challenges involved. Moreover, emphasizing digital resilience in interventions ensures that forced migrants are not only able to navigate current digital health systems but are also prepared to adapt to future technological advancements, thereby fostering their long-term empowerment and inclusion.

### Conclusions

This study highlights the crucial importance of improving DHL among forced migrant populations, a need that has intensified in a global context marked by the accelerated digitalization of health care services. The results of our research revealed that, while the interventions in most of the studies (5/6, 83%) were effective, significant challenges persist, requiring targeted and context-adapted strategies. The positive effects observed underscore the potential of culturally and linguistically adapted interventions to improve the health and well-being of forced migrant populations.

However, addressing these challenges requires more than isolated actions; it necessitates a collaborative and multisectoral approach that ensures that interventions, whether they involve training, social support, or technological resources, are contextually adapted. Developing innovative solutions tailored to the specific needs of forced migrant populations remains essential to strengthen their DHL. These efforts must rely on integrated approaches, bringing together health care systems, service providers, technology developers, decision-makers, and community organizations. To ensure the effectiveness of interventions, future research must prioritize rigorous methodologies, including a systematic evaluation of DHL levels before and after interventions, as well as greater diversity and representativeness of the studied samples.

Promoting DHL among forced migrant populations is not only a public health imperative but also a societal issue. It serves as a cornerstone for their successful integration into host societies, the optimization of health care services, and the reduction of health inequalities. By investing in inclusive and evidence-based interventions, it is possible to improve DHL levels across populations, including forced migrants. Such initiatives not only address the immediate needs of forced migrant populations but also pave the way for a more equitable, accessible, and resilient society for all. Through collective effort and shared commitment, the promise of digital health can become a reality for everyone, regardless of origin or circumstances.

## Appendix

Multimedia Appendix 1 PRISMA (Preferred Reporting Items for Systematic Reviews and Meta-Analyses) 2020 checklist guidelines for systematic reviews.

Multimedia Appendix 2 Eligibility criteria.

Multimedia Appendix 3 Search strategies for each database.

## Abbreviations

DHL: digital health literacy
HLS-EU-Q16: European Health Literacy Survey Questionnaire
LEP: limited English proficiency
PICOS: population, intervention, comparison, outcomes, and study design
PLN: peer language navigator

## Data Availability

All data generated or analyzed during this study are included in this published paper (and its supplementary information files). The databases created during the data extraction process are available from the corresponding author on reasonable request.
