# Supplementary material for: Effectiveness of Interventions to Improve Digital Health Literacy in Forced Migrant Populations: Mixed Methods Systematic Review
Source: J Med Internet Res. 2025 Jun 25;27:e69880. doi: 10.2196/69880 (PMC12242062; doi:10.2196/69880)
Supplement: Multimedia Appendix 2 [file jmir_v27i1e69880_app2.docx]

**Appendix 2: Eligibility criteria based on the PICOS (population, intervention, comparison, outcomes, and setting or context) model [1,2,3].**

| PICOS model categories | Description |
| --- | --- |
| Population (P) | The population consists of forced migrant populations, including internally displaced persons, refugees, and asylum seekers, as well as political, economic, and climate migrants. It should be noted that forced migrant populations research is challenged by the diversity of terminology and definitions used [2]. For this systematic review, we will rely on definitions from the International Organization for Migration [1] glossary, as follows:   - Internally displaced persons are “persons forced to flee or leave their homes or places of habitual residence within their own country, including as a result of or to prevent the effects of conflict, violence, human rights violations or natural or man-made disasters” (free translation) [1]. - Refugees (convention 1951) means any “person who, owing to a well-founded fear of being persecuted for reasons of race, religion, nationality, membership of a particular social group or political opinion, is outside the country of his nationality and who is unable or, owing to such fear, does not want to claim the protection of this country” (free translation) [1]. - An asylum seeker is a “person seeking international protection. In countries with individualized examination procedures, the asylum-seeker is a person whose asylum application has not yet been the subject of a final decision by the potential host country. Not every asylum seeker is necessarily recognized as a refugee at the end of the process, but every refugee has initially been an asylum seeker” (free translation) [1]. - Migrants include all persons who leave their place of habitual residence to settle, temporarily or permanently, either in another region within the same country or in another country, thus crossing an international border, and for various reasons [1]. When people move in search of a better life or work, we speak of economic migration. In addition, when they migrate to escape persecution due to their political opinions, it is referred to as political migration [1]. - Climate migration means any “movement of a person or group of persons who, essentially for reasons related to a sudden or gradual change in the environment as a result of climate change, are forced to leave their place of habitual residence or leave it on their own initiative, temporarily or permanently, to go elsewhere in the territory of a State or across an international border” (free translation) [1].   All studies on interventions related to promoting digital health literacy among forced migrant populations will be included. |
| Intervention (I) | - All studies on interventions related to promoting digital health literacy among forced migrant populations will be included. - All types of interventions will be considered and classified according to the “Behaviour Change Wheel” model [3]. Indeed, this model makes it possible to systematically characterize interventions aimed at modifying or changing behavior at the individual, organizational, and societal levels. |
| Comparative (C) | There will be 2 types of comparisons:   - A specific intervention to promote digital health literacy in forced migrant populations, compared to no specific intervention or usual services - A specific intervention to promote digital health literacy in forced migrant populations, compared to any other intervention to promote forced migrant populations in these populations |
| Outcomes (O) | The following elements will be examined:   - Categories: the following categories will be reviewed: - Level of intervention: individual, group, or mixed - Mode of design: theory or evidence or none - Targeted behavior: opportunities, motivations, attitudes, and abilities or skills - Characteristics of interventions: interventions will be classified according to the following 9 functions of an intervention (ie, types of intervention) according to the model proposed by Michie et al [3]: education, persuasion, incentive, coercion, training, restriction, environmental restructuring, modeling, and empowerment   Results and success factors of interventions: outcomes related to behavior change, self-care behaviors, and resolution of health problems through interventions will be examined along with their success factors. We will also explore outcomes related to access to basic health information and improved quality of life through digital health technologies |
| Study design (S) | As for the types of studies, there will be no restrictions. All quantitative empirical studies, qualitative studies, or mixed methods studies, and studies with or without a control group will be included without distinction. |

References

1. Organisation internationale pour les migrations (OIM). Termes clés de la migration 2021 [Available from: https://www.iom.int/fr/termes-cles-de-la-migration.

2. Keely CB, Kraly EP. Concepts of Refugee and Forced Migration: Considerations for Demographic Analysis. In: Hugo G, Abbasi-Shavazi MJ, Kraly EP, editors. Demography of Refugee and Forced Migration. Cham: Springer International Publishing; 2018. p. 21-37.

3. Michie S, van Stralen MM, West R. The behavior change wheel: A new method for characterizing and designing behavior change interventions. Implementation Science. 2011;6(1):42.
